# Disease of the Lining Membrane of the Ear

**Published:** 1848-04

**Authors:** 


*Disease of the Lining Membrane of the Ear, followed by Purulent Effusion within the Skull.*—Dr. Hutton communicated to the Dublin Pathological Society, a case of disease of the internal ear. The subject was a boy, nine years of age, who for a long period had a discharge from the left ear. Ten days before his admission into the Richmond Hospital, the discharge from the ear was almost completely suppressed; he vomited and had fever; got gradually worse, and was brought to the hospital. On his admission, he had the head retracted; any effort to restore it to the natural position caused great pain; the muscles of the back of the neck were spasmodically affected; the general aspect ghastly, the eyes straining, the teeth uncovered by the lips, and the whole body emaciated. He understood questions and returned answers, but in a few hours afterwards fell into a state of coma, and died. In examining the body after death, the external opening of the affected ear was found filled with caseous matter. There was an opening in the membrana tympani, and at the insertion of the membrana there was a fungous growth; the mucous membrane lining the cavity of the tympanum was thickened and granular, and adhered firmly to the bones; the ossicula remained. The temporal bone itself was not carious nor softened, nor was its mastoid process at all diseased. The dura mater was separated from the petrous portion, and there was lymph effused between them; the dura mater was sloughy, and there was an aperture in it. The upper surface of the cerebellum was smeared with purulent matter; the inferior surface of the sensorium was similarly smeared; the brain was not softened; no communication could be traced between the cavity of the cranium and the internal ear.—*Abridged from Dub. Jour.*

